# Transoral vertebroplasty of the lateral mass of C1 using image guidance

**DOI:** 10.1007/s00701-017-3158-4

**Published:** 2017-04-03

**Authors:** Pujan Kavakebi, P. P. Girod, S. Hartmann, A. Tschugg, C. Thomé

**Affiliations:** Department of Neurosurgery, Medical University of Innsbruck, Anichstrasse 35, 6020 Innsbruck, Austria

**Keywords:** Transoral, Image guidance, Navigation, Atlas, C1, Vertebroplasty

## Abstract

**Background:**

Osteolytic lesions of the anterior aspects of C1 (lateral mass) are difficult to address in a minimally invasive fashion and are often treated by craniocervical instrumentation.

**Methods:**

We report the feasibility and technical method of transoral vertebroplasty of the lateral mass of the atlas using image guidance and describe the workflow of the procedure. To our knowledge, there has not yet been a technical description of a transoral vertebroplasty using image guidance.

**Results:**

Adequate positioning of the pedicle access needle using image guidance for addressing the lateral mass of C1 through a transoral, permuceous access can be achieved.

**Conclusions:**

With the assistance of image guidance, it is safe and feasible to access the lateral mass of the atlas. This constitutes a minimally invasive and fast alternative for introducing the bone needle to C1 rather than using a fluoroscopic device alone.

## Introduction

Vertebroplasty of osteolytic lesions in metastatic spinal disease is a widely accepted technique in the treatment of painful lesions and can reduce the possibility of a fracture in the affected vertebral body by increasing stability [3, 8]. Vertebroplasty is mostly used in the thoracolumbar spine where access of the vertebral body via the pedicle has been proven to be safe and efficient. On the contrary, this technique is not (frequently) used in the upper spine. Furthermore in C1, fluoroscopic guidance alone is not safe enough because of possible life-threatening complications. A minimally invasive approach using image guidance could be an attractive alternative.

## Materials and methods

The image guidance setup at our department consists of a combination of an intraoperative CT (SOMATOM Definition AS, Siemens Healthcare GmbH, Erlangen, Germany) and the Spine & Trauma 3D Navigation software with the corresponding navigation system (Brainlab AG, Feldkirchen, Germany). The patient lays supine and is placed in a Mayfield radiolucent skull clamp using radiolucent head pins to avoid artifacts. In a transoral setup the spinal reference array cannot be attached to the spine. Thus, the cranial reference array is attached to the reference adapter arm for the radiolucent Mayfield for cranial surgery. A CT angiography is performed prior to draping and the automatic image registration is performed following the Spine & Trauma 3D software walkthrough. Alternatively, the intraoperative CT can be fused with the pre-operative CT angiograms. A diamond tip (3.5 mm) referenced pedicle access needle (DePuy Synthes, Zuchwil, Switzerland) is calibrated with the ICM4 (Instrument Calibration Matrix, Brainlab AG).

After performing the CT, a Boyle–Davis mouth gag is used to expose the pharyngeal wall, which is washed with antiseptic solutions. The spinal software requires an accuracy check and for this purpose the teeth are the best anatomical structure.

**Fig. 1:**
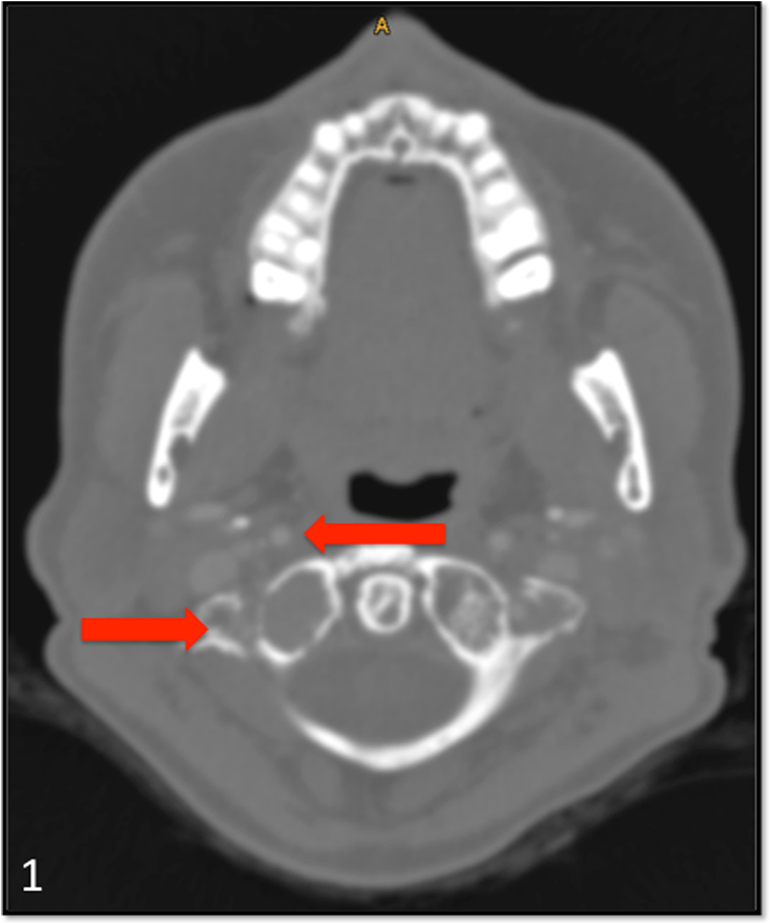
CT angiogram of an osteolytic lesion in the right lateral mass of the atlas in a patient with multiple myeloma. *Arrows* show the internal carotid and vertebral arteries

**Fig. 2:**
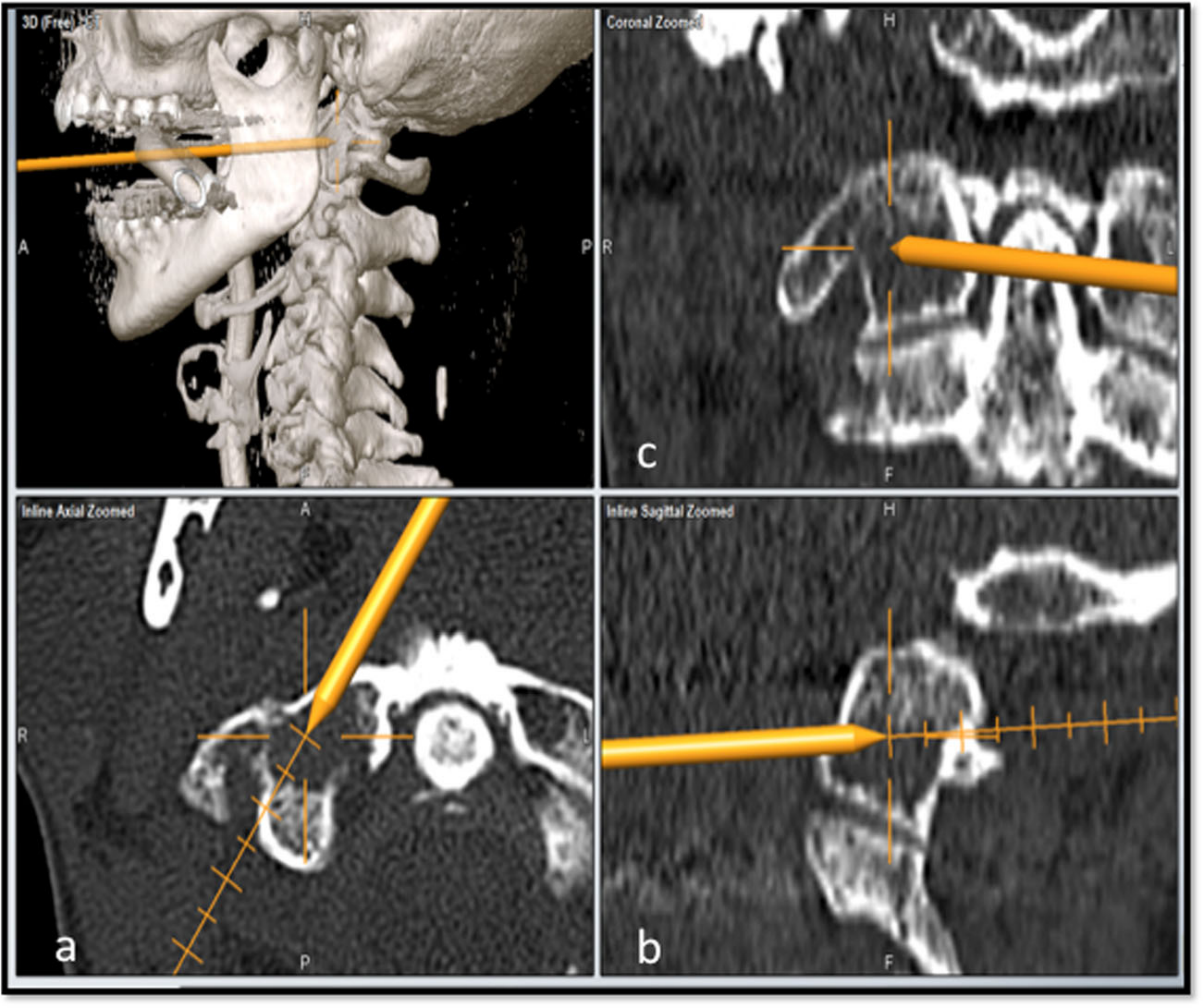
Snapshot of the navigation monitor in a multiplanar 3D view showing the bone needle inside the osteolytic lesion [axial (**a**), sagittal (**b**), coronal (**c**)]

The pedicle access needle is then used to address the lateral mass of the atlas by entering the pharyngeal wall from the midline to avoid injury to the internal carotid artery and the palatine tonsil (Fig. 1). With the help of image guidance and a multiplanar 3D reconstructed view, the craniocaudal and lateral projection is set and the needle gently introduced permucosal into the lateral mass of C1 until a final position in the mid of the osteolytic lesion is reached. By virtual vision, harm to the vertebral artery can be avoided (Fig. 2).

**Fig. 3:**
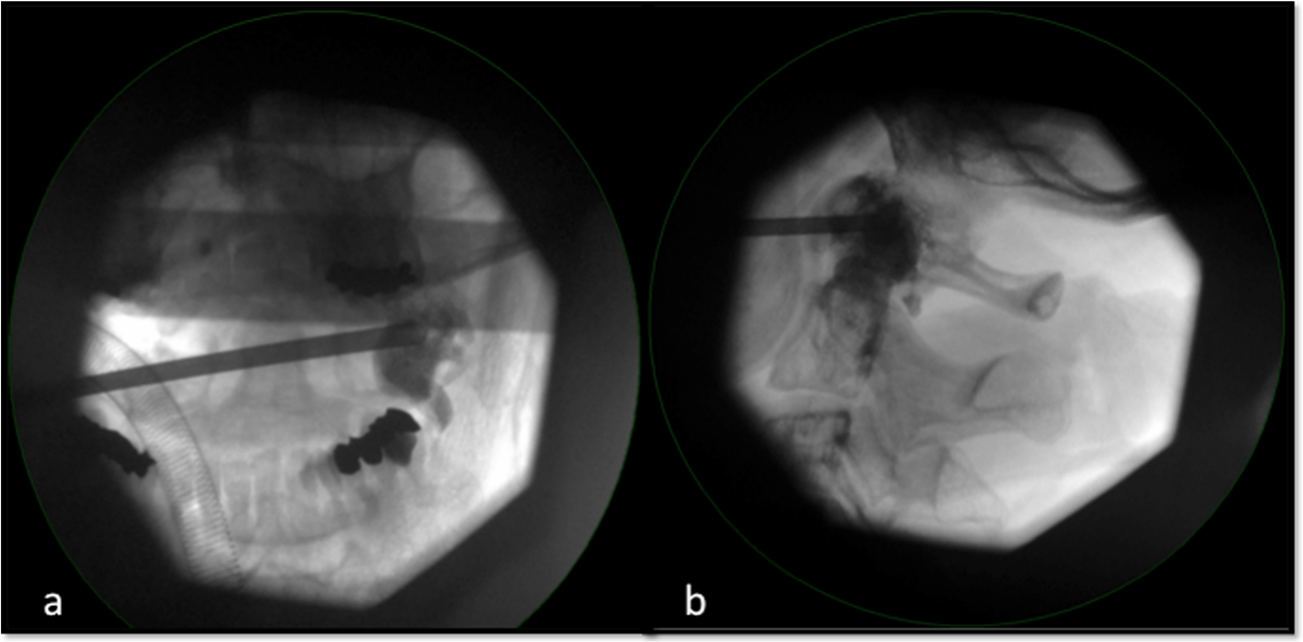
Fluoroscopic AP (**a**) and lateral (**b**) view while introducing the high-viscosity bone cement

Bone cement with high viscosity is introduced into the osteolytic lesion under fluoroscopic control (Fig. 3). After filling the lesion with approximately 2 ml of bone cement, the needle is removed and the wound is closed with a self-resorbable suture.

**Fig. 4:**
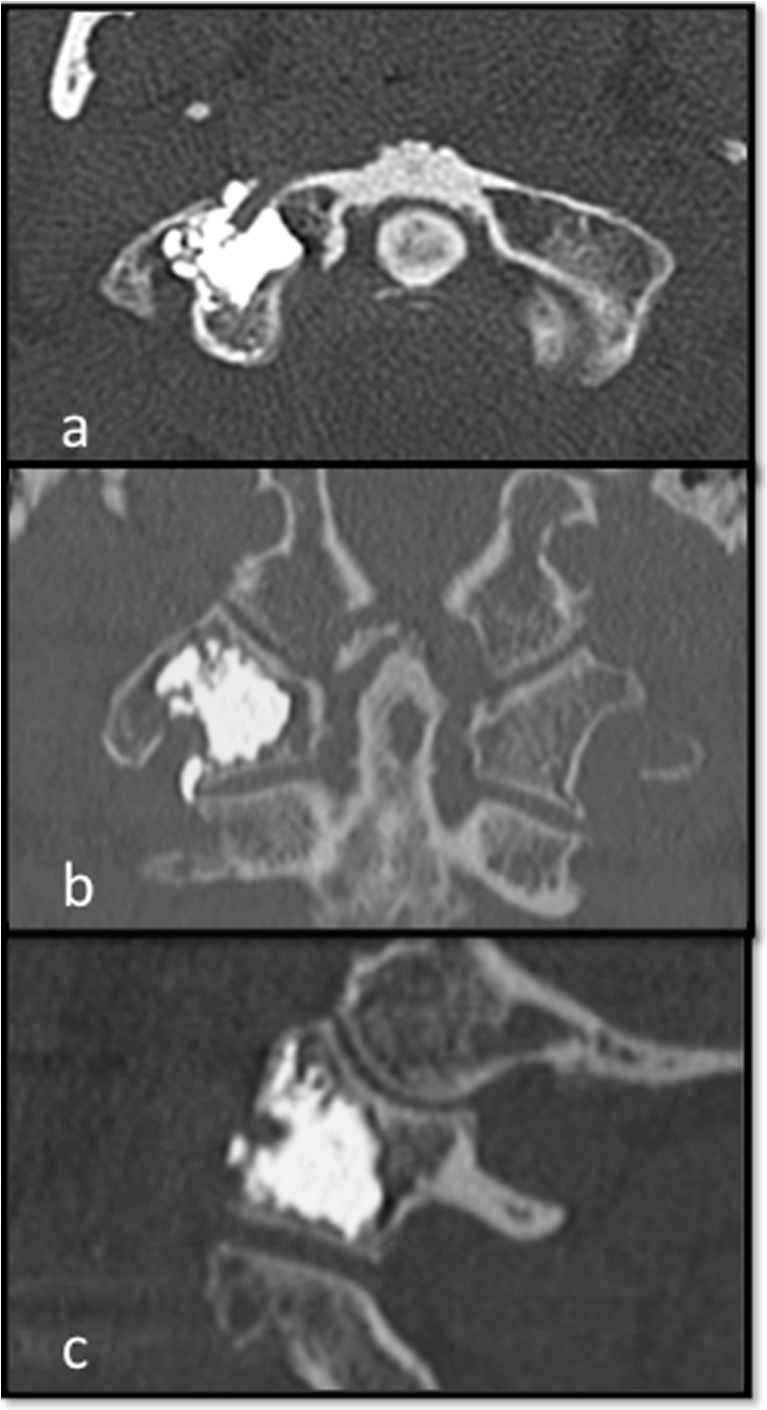
Intraoperative CT after cement augmentation [axial (**a**), coronal (**b**), sagittal (**c**)]

The cement augmentation is then checked with an intraoperative CT of just the field of interest in reduced radiation mode (Fig. 4).

Four patients have been treated with this navigational setup through a transoral/transnasal access so far without procedural complications (Table 1).

**Table 1:** ᅟ

*n*	Sex/age	Indication	Procedure	Access	Procedural complication
1	f/58	Osteolysis C1/multiple myeloma	Cement augmentation C1	Transoral	None
2	f/30	Benign cyst in C2	Resection + iliac strut + C1/C2 fixation	Transoral	None
3	m/50	Basilar invagination	Transnasal resection of C2 tip + C0-C3 fixation	Transnasal	None
4	f/78	Osteolysis C2/metastasis	Cement augmentation C2	Transoral	None

## Results

Adequate positioning of the pedicle access needle using image guidance for addressing the lateral mass of C1 through a transoral, permuceous access can be achieved.

## Discussion

Metastatic spine disease is a common presentation in high-volume spine centers. These patients frequently suffer from painful osteolytic lesions that require vertebroplasty for pain relief and stabilization of the vertebral body. Most lesions are located in the thoracic and lumbar spine, but in rare cases the cervical spine, especially the upper cervical spine, may be affected [6]. These patients suffer from severe pain in the craniocervical junction. Additionally, the risk of instability due to possible fractures affects their quality of life.

Aggressive treatment by craniocervical fixation is often not the goal in progressive tumor disease and therefore transoral permucosal vertebroplasty represents a safe and effective alternative in the palliative setting.

The open transoral/transpharyngeal approach could be shown as a safe technique, which is rapid and effective. Postoperative complications are rare, but infections or velopalatine incompetence may occur [7].

In addition, a lateral access to C1 and C4 using a combination of CT and fluoroscopic guidance was described previously [4]. An anterolateral percutaneous approach to C2 and a transoral approach for vertebroplasty of C2 were described in 2002 [1, 5, 9]. The first publication of a vertebroplasty via a transoral access to C1 was published in 2013 [2]. The group showed a case series of patients presenting with osteolytic tumor lesion in C1. The aim was to gain stability and pain relief. They used a fluoroscopic guidance and an intraoperative CT for checking the needle position.

A transoral permucosal targeting of the lateral mass of the atlas can be challenging when performed by fluoroscopic guidance only because visibility of the internal carotid and vertebral artery is only possible by using a simultaneous angiography. On the other hand, no classic percutaneous targeting technique using the fluoroscopic devise exists in this region of interest.

Therefore, we have introduced a new image guidance technique to address this problem by combining the cranial navigation hardware (reference array attached to the reference adapter arm of the Mayfield clamp) with the spinal navigation software (Spine & Trauma 3D, Brainlab). The lateral mass of C1 is a small anatomic region surrounded by vital structures. A precise positioning of the bone needle is therefore essential to minimize the risk of violating vessels and cement leakages.

Then high-resolution image guidance in a multiplanar reconstruction can be very helpful for accuracy and reduces the amount of radiation to the medical staff. A standardized protocol for transoral approaches using image guidance was established. By performing CT angiograms or image-fusion of pre-operative CTA with intraoperative CT, data visualization of vital vessel structures can be achieved and violation may be avoided. This procedure can be used simultaneously and bilaterally in C1 and C2, if necessary. Alternatively, a posterior transcutaneous approach to the lateral mass of C1 can be used, but bears the risk of harming the vertebral artery or the exiting nerve root under the C1 arch.

Contraindications for the transoral, permuceous access to C1 are dental sepsis and poor fluoroscopic visibility to the cortical borders of C1 during the cement application.

## Conclusions

Image-guided transoral vertebroplasty constitutes a safe and minimally invasive approach to osteolytic destructions of the C1 lateral mass.
